# Immediate effects of muscle energy technique on pain and range of motion in patients with chronic non-specific neck pain: A randomized controlled trial

**DOI:** 10.12669/pjms.41.5.9601

**Published:** 2025-05

**Authors:** Ghulam Hussain, Aatik Arsh, Fazal Rehman, Shumaila Khan

**Affiliations:** 1 Ghulam Hussain, DPT, MSPT. Physical Therapy Department, Mardan Medical Complex MTI-MMC, Mardan, Pakistan; 2 Aatik Arsh, DPT, MSPT, PhD. Institute of Physical Medicine and Rehabilitation, Khyber Medical University, Peshawar, Pakistan; 3 Fazal Rehman, DPT, MSPT. Physical Therapy Department, Khyber Teaching Hospital, MTI-KTH, Peshawar, Pakistan; 4 Shumaila Khan, DPT, MSPT. Office of Research, Innovation and Commercialization, Khyber Medical University, Peshawar, Pakistan

**Keywords:** Exercises, Manual Therapy, Neck Pain, Physical Therapy, Rehabilitation

## Abstract

**Objective:**

To determine the immediate effects of muscle energy technique and conventional physical therapy compared to conventional physical therapy alone on pain and range of motion (ROM) in patients with chronic mechanical neck pain.

**Methods:**

A randomized controlled trial (RCT) was conducted at Physical Therapy Department Hayatabad Medical Complex Peshawar from March 2022 to August 2022. A total of 50 subjects, aged 18 to 50 years with non-specific neck pain for ≥ 3 months were included. Participants were randomly assigned to two groups through lottery approach. Participants in experimental group (n=25) received muscle energy technique along with conventional physical therapy while participants in control group (n=25) received conventional physical therapy. Baseline and post-intervention (immediately after the session) neck pain intensity and neck ranges were measured by Numeric Pain Rating Scale and goniometer respectively.

**Results:**

Mean age of participants was 32.30 ± 7.75. Of the 50 participants, 36 (72%) were male while the remaining 14 (28%) participants were female. At baseline, there were no significant differences (P>0.05) between experimental and control groups. However, at post-intervention, there were significant differences in pain intensity (p=0.024), cervical flexion (p<0.001), extension (p=0.003), left rotation (p<0.001), right rotation (p<0.001), left lateral flexion (p=0.027) and right lateral flexion (p<0.001) ROMs between experimental and control groups.

**Conclusion:**

Muscle energy technique along with conventional physical therapy is more effective in decreasing neck pain and improving cervical ROM compared to conventional physical therapy alone in individuals with chronic mechanical neck pain.

***Trial Registration:*** Iranian Registry of Clinical Trials (Reference No.: IRCT20220209053976N1).

## INTRODUCTION

Neck pain is a common disabling musculoskeletal condition that can lead to decrease in working productivity, debility, and substantial financial costs.^1^ About one-third adults experience neck pain annually and about 5–10% cause significant disability. It is reported that neck pain is the 4^th^ leading cause of disability-adjusted life years (DALYs) globally.^2^,^3^ There is a scarcity of high-quality studies which reported epidemiology of neck pain in Pakistan, however, it is reported that prevalence of neck pain is high in Pakistan and is a major socioeconomic burden.^4^,^5^

A wide range of physical therapy techniques can be used to manage neck pain including massage therapy, nerve mobilization or nerve tissue management, changes in pillow height, applying kinesiology tapping, heat therapy, and ergonomic education.^6^ For chronic neck pain, cervical, scapular and thoracic region manual therapy have been reported to be effective.^7^ One of the manual therapy technique to decrease cervical pain and increase neck range of motion (ROM) is muscle energy technique (MET). MET is a procedure of manual or ‘hands-on’ therapy applied by osteopathic physicians, chiropractors, and physical therapists. MET uses the patient’s own voluntary muscular contraction in an accurately controlled direction against an operator counter applied force.^8^ It is originally an osteopathic manipulation method in which patient actively contract muscles (isometric/isotonic) to improve function of musculoskeletal system and decrease pain.^9^,^10^

Evidence suggests that MET is effective in managing neck pain,^11^ however, there is a scarcity of studies that assessed the immediate effects of MET in individuals with neck pain. Humans tend to search for fast and effective results and in this regard immediate effects of manual therapy techniques need to be explored. Therefore, current study was designed to determine the immediate effects of muscle energy technique and conventional physical therapy compared to conventional physical therapy alone on pain and ROM in patients with chronic mechanical neck pain.

## METHODS

A randomized controlled trial (RCT) was conducted at Physical Therapy Department Hayatabad Medical Complex Peshawar from March 2022 to August 2022. Male and female subjects aged 18 to 50 years with non-specific neck pain for ≥ 3 months were included. Non-specific neck pain was defined as pain in the cervical region that cannot be attributed to a specific underlying pathology and is associated with poor posture, muscle strain, or minor mechanical dysfunction.^4^ Patients with cervical radiculopathy or disc bulge, trauma to cervical spine, structural spinal deformity, osteoporosis, instability, fracture, joint dislocation, dermatitis, active bleeding, open wounds, neck surgery in the past 12 months, and malignancy were excluded from the study. Potential participants were identified with help of physical therapists working at the study site. Subjective and objective examination was performed to confirm the diagnosis of chronic non-specific neck pain. All the eligible participants were comprehensively assessed by physical therapists with at least five years of clinical experience. Participant information sheets were provided to potential participants and if they were willing to participate in the study, written informed consent was obtained from each participant.

### Ethical Approval:

It was obtained from ethics committee IPM&R Khyber Medical University Peshawar (Reference No.: DIR/IPM&R-EC/2201 dated February 25, 2022. The trial was prospectively registered with Iranian Registry of Clinical Trials (Reference No.: IRCT20220209053976N1).

Sample size was calculated using G*Power Software. A total of 50 participants (25 in each group) were required to conduct the study (Effect size d = 1.05254, α error probability = 0.05, power (1-β error probability) = 0.95). Effect size was calculated based of the findings of previous study.^12^ Participants were randomly assigned to two groups through lottery approach. Participants in control group (n=25) received conventional physical therapy. Conventional physical therapy included heating pad for 15-20 minutes and soft tissue release in cervical region for five minutes. Participants in experimental group (n=25) received MET along with conventional physical therapy. MET was applied by a musculoskeletal physical therapist with at least five years of clinical experience. Patients applied resistance to therapists to produce isometric contraction in the muscles just short of the barrier. The patient strength of muscle contraction was less than the therapist counter force of resistance. Therapist held the position for 7-10 seconds. Then a 30 second stretch was given, and the process was repeated three times bilaterally. MET was applied to each specific muscle as described below:

### Upper trapezius:

Patient was in supine lying. Therapist one hand on shoulder and other on occiput. Patient neck was in contralateral side flexion and full contralateral, partial contralateral and slight ipsilateral rotations for posterior, middle and anterior fibers respectively. MET was applied.

### Levator scapulae:

Patient was lying supine with therapist one hand on shoulder and the other on occiput. Patient head was in flexion, contralateral side flexion and contralateral rotation. MET was applied.

### Sternocleidomastoid:

Patient was supine with towel under shoulder to keep neck in 10-15 degrees extension. Therapist kept one hand on sternum and other on occiput. Therapist brought patient neck into contralateral side bending and contralateral rotation. Patient was asked to lift head slightly towards ceiling and maintain that position for 7-10 seconds against gravity resistance followed by 30 seconds stretch with three repetitions.

### Scalene muscles:

Patient was in supine position with towel under thoracic area to cause slight neck extension. Therapist brought patient neck into contralateral side flexion and full, mid and slight contralateral rotations for posterior, middle and anterior scalene respectively. Therapist placed one hand on occiput while the other hand was on second rib below lateral clavicle, on second rib below center of clavicle and on the sternum for posterior, mid and anterior scalene respectively. MET was applied.^13^

Baseline and post-intervention (immediately after the session) neck pain intensity and neck ROM were measured by Numeric Pain Rating Scale and goniometer respectively.^4^ The current study was a single blinded study and assessors who collected data at baseline and post-intervention were blind to the allocation. Due to the nature of the study, it was not possible to blind participants and therapists. Data analysis was performed using SPSS version 28. Normality of data was assessed using Shapiro-Wilk test. As data was normally distributed, therefore independent sample T-test was used for between group comparisons.

## RESULTS

Mean age of participants was 32.30 ± 7.75. Of the 50 participants, 36 (72%) were male while the remaining 14 (28%) participants were female. There were 25 participants in each group, (Table-I). At baseline, there were no significant differences (P>0.05) in neck pain and ROM between experimental and control groups, (Table-II).

**Table-I:** Demographic data of participants in control and experimental groups

Variables	CG (% / M ± SD)	EG (% / M ± SD)	Total
Age	33.08 ± 8.09	31.52 ± 7.49	32.30 ± 7.75
Gender			
Male	19 (76%)	17 (68%)	36 (72 %)
Female	6 (24%)	8 (32%)	14 (28 %)
Neck Pain Duration			
3-6 months pain	8 (32%)	8 (32%)	16 (32 %)
6-12 months pain	7 (28%)	8 (32%)	15 (30 %)
1-2 years pain	6 (24 %)	5 (20%)	11 (22 %)
More than 2 years pain	4 (16%)	4 (16%)	8 (16 %)

CG: Control Group, EG: Experimental Group, M: Mean, SD: Standard Deviation.

**Table-II:** Baseline pain and neck ROM of experimental and control groups

Variable	CG (M ± SD)	EG (M ± SD)	P – value
Baseline Pain Intensity	6.36 ± 1.07	6.56 ± 1.15	0.566
Baseline Neck Flexion	31.08 ± 4.89	31.64 ± 6.95	0.743
Baseline Neck Extension	34.28 ± 4.80	33.64 ± 5.57	0.669
Baseline Neck Left Rotation	34.28 ± 5.45	34.80 ± 9.75	0.817
Baseline Neck Right Rotation	35.60 ± 6.59	33.80 ± 7.53	0.373
Baseline Neck Left Lateral Flexion	34.00 ± 4.42	32.84 ± 5.11	0.395
Baseline Neck Right Lateral Flexion	33.96 ± 5.65	33.96 ± 6.01	1.000

CG: Control Group, EG: Experimental Group, M: Mean, SD: Standard Deviation.

However, at post-intervention, there were significant differences in pain intensity (p=0.024), cervical flexion (p<0.001), extension (p=0.003), left rotation (p<0.001), right rotation (p<0.001), left lateral flexion (p=0.027) and right lateral flexion (p<0.001) ROMs between experimental and control groups, (Table-III).

**Table-III:** Post-Intervention pain and neck ROM of experimental and control groups

Variable	CG (M ± SD)	EG (M ± SD)	P – value
Post–Intervention Pain Intensity	3.60 ± 1.04	2.84 ± 1.21	0.024
Post–Intervention Neck Flexion	33.44 ± 5.06	39.20 ± 4.69	<0.001
Post–Intervention Neck Extension	36.08 ± 4.62	40.40 ± 4.94	0.003
Post–Intervention Neck Left Rotation	36.20 ± 5.46	43.08 ± 8.60	<0.001
Post–Intervention Neck Right Rotation	37.88 ± 6.48	42.44 ± 6.38	<0.001
Post–Intervention Neck Left Lateral Flexion	36.44 ± 4.74	39.64 ± 5.17	0.027
Post–Intervention Neck Right Lateral Flexion	36.56 ± 5.77	41.00 ± 4.79	<0.001

CG: Control Group, EG: Experimental Group, M: Mean, SD: Standard Deviation.

## DISCUSSION

Neck pain a common musculoskeletal condition, affecting people around the globe, often causing physical impairments and disability.^14^ The present study investigated the immediate effects of muscle energy technique (MET) combined with conventional physical therapy compared to conventional physical therapy alone on pain intensity and cervical ROM in patients with chronic non-specific neck pain. Our findings demonstrated that MET in combination with conventional therapy significantly reduced pain intensity and improved cervical ROM, including flexion, extension, lateral flexion, and rotation, when compared to conventional therapy alone. These results highlight the efficacy of MET as an adjunct therapy for individuals with chronic neck pain.

There is paucity of literature which assessed immediate effects (directly after the intervention session) of MET in individuals with neck pain, however, there are some studies which assessed immediate effects of other physical therapy techniques and in accordance with the findings of current study, previous studies reported that physical therapy techniques can alleviate pain immediately.^15^,^16^ A study reported that manual therapy improved neck pain and ROM immediately and at follow ups.^17^ Another study reported that single application of techniques like trigger point ischemic compression on upper trapezius having latent myofascial trigger points resulted in ROM improvement and pain reduction immediately after session.^18^ Despite this, majority of the previous studies assessed effects of physical therapy techniques after application of the technique for specific duration (from two weeks to three months) in the management of neck pain. Generally, it is argued that physical therapy techniques including MET should be applied for two weeks (14 days) to achieve maximum benefits from the specific physical therapy technique.^16^,^19^

Although literature is scarce regarding the immediate effects of MET, however, positive short-term effects of MET after application of MET for specified time are well established. A recent systematic review reported that MET has significant effects in managing acute and chronic non-specific mechanical neck pain. The review concluded that MET is better choice to treat acute or chronic non-specific neck pain disorders. Moreover, the review suggested that MET is more effective in combination with other conventional treatments of rehabilitation.^11^ Another study also reported that MET have better results in the management of neck dysfucntion.^20^

The positive effects of MET are not specific to patients with neck pain, but it also has positive effects in reducing pain and improving ROM in patients with other musculoskeletal conditions. Evidence suggests that MET can be applied in patients with wide range of muscoloskeltal conditions including neck pain, low back pain and shoulder pain.^8^,^10^,^21^ The findings of current study and previous studies suggest that it is imperative for physical therapists to utilize MET along with other conventional physical therapy techniques in the management of muscoloskeltal conditions of mechanical origin. MET may not only help is managing the symptoms associated with the muscoloskeltal conditions but may also assist in correcting the biomechanical parameters.^22^,^23^

One of the novel contributions of this study is the emphasis on immediate post-intervention effects, which has been relatively underexplored in previous literature. While many studies focus on long-term rehabilitation outcomes, our study provides valuable insights into the rapid effectiveness of MET in clinical settings. This is particularly relevant for patients seeking quick relief from neck pain, such as office workers, athletes, and individuals with postural dysfunctions. Additionally, our study contributes to the growing body of evidence supporting the integration of MET into conventional physiotherapy regimens for neck pain management.

### Strength of study:

The strengths of our study include its randomized controlled design, the use of standardized assessment tools (Numeric Pain Rating Scale and goniometer), and the inclusion of blinded assessors to minimize bias. Furthermore, the intervention was delivered by experienced musculoskeletal physical therapists, ensuring consistency in technique application.

### Limitations:

In current study, only assessor were blind, and therapists and participants were aware of the allocation, therefore the findings may need to be interpreted with caution. As the objective of current study was to assess only the immediate effects (after one session only) of MET, therefore we were not able to comment on the long-term effects of MET is patients with chronic mechanical neck pain.

## CONCLUSION

The study concluded that MET along with conventional physical therapy is more effective in decreasing neck pain and improving cervical ROM immediately compared to conventional physical therapy alone in individuals with chronic mechanical neck pain. Large, multisite clinical trials to assess the short- and long-term effects of MET are recommended.

### Authors’ contributions:

**GH:** Conceived and designed the study, collected the data, performed the analysis and prepared the manuscript.

**AA:** Conceived and designed the study, performed the analysis and prepared the manuscript.

**FR, SK:** Performed literature review, Collected the data, Performed Critical Review.

All authors are responsible for the integrity of the study. All authors have read and approved the final manuscript.
